# Seaweed Blends as a Valuable Source of Polyunsaturated and Healthy Fats for Nutritional and Food Applications

**DOI:** 10.3390/md19120684

**Published:** 2021-11-30

**Authors:** Francisca Marques, Diana Lopes, Elisabete da Costa, Tiago Conde, Andreia Rego, Ana Isabel Ribeiro, Maria Helena Abreu, Maria Rosário Domingues

**Affiliations:** 1 ALGAplus—Production and Trading of Seaweed and Derived Products Lda., 3830-196 Ílhavo, Portugal; francisca.marques@ua.pt (F.M.); andreia.rego@algaplus.pt (A.R.); ana.ribeiro@algaplus.pt (A.I.R.); helena.abreu@algaplus.pt (M.H.A.); 2 Mass Spectrometry Centre, LAQV-REQUIMTE, Department of Chemistry, University of Aveiro, Santiago University Campus, 3810-193 Aveiro, Portugal; dianasalzedaslopes@ua.pt (D.L.); elisabetecosta@ua.pt (E.d.C.); tiagoalexandreconde@ua.pt (T.C.); 3 Centre for Environmental and Marine Studies, CESAM, Department of Chemistry, University of Aveiro, Santiago University Campus, 3810-193 Aveiro, Portugal

**Keywords:** seaweed blends, chemical composition, fatty acids, nutritional indices, lipidomics

## Abstract

Seaweeds are considered healthy and sustainable food. Although their consumption is modest in Western countries, the demand for seaweed in food markets is increasing in Europe. Each seaweed species has unique nutritional and functional features. The preparation of blends, obtained by mixing several seaweeds species, allows the obtaining of maximum benefits and ingredients with single characteristics. In this work, five seaweed blends, commercially available and produced under organic conditions in Europe, were characterized. The proximal composition included contents of ash (20.28–28.68% DW), proteins (17.79–26.61% DW), lipids (0.55–1.50% DW), and total carbohydrates (39.47–47.37% DW). Fatty acid profiles were determined by gas chromatography–mass spectrometry (GC–MS), allowing quantification of healthy fatty acids, namely *n*-3 and *n*-6 polyunsaturated fatty acids (PUFA), and calculation of lipid quality indices. Each blend showed a characteristic PUFA content in the lipid pool (35.77–49.43% of total fatty acids) and the content in essential and healthy *n*-3 PUFA is highlighted. The atherogenicity (0.54–0.72) and thrombogenicity (0.23–0.45) indices evidenced a good nutritional value of lipid fractions. As nutritional and environmentally attractive products, the consumption of the studied seaweed blends can contribute to a healthy lifestyle.

## 1. Introduction

The consumption of seaweeds has been widespread in Asia for centuries, where they are consumed as food and also used in traditional medicine and in dietary therapies [1]. Indeed, seaweeds have an unparalleled richness in nutrients and bioactive compounds, including bio-available vitamins, minerals, pigments, proteins, bioactive peptides, dietary fiber, lipids and phytochemicals [2,3,4]. Thus, in recent years, their consumption has increased in Western countries. Seaweeds are considered a sustainable and healthy food [5], associated with beneficial effects, including the prevention of non-communicable diseases (NCDs) as cardiovascular diseases, cancers, and diabetes [6]. 

Seaweeds, also known as marine macroalgae, are photosynthetic organisms classified into three phyla, Chlorophyta (green), Rhodophyta (red) and Ochrophyta (brown), which show great variability between their chemical compositions [7,8]. Even though the lipid fraction of seaweeds represents only 1–6% of dry weight (DW) [9,10,11,12,13,14,15], seaweeds are an important source of fatty acids, including healthy polyunsaturated fatty acids (PUFA). In general, seaweeds are characterized by a fatty acid composition that is quite advantageous for human health, with amounts of unsaturated fatty acids (UFA) usually higher than the amounts of saturated fatty acids (SFA) [7,16], which are negatively associated with the occurrence of NCDs [17]. High concentrations of PUFA in the lipid fractions have been reported in several seaweed species, such as *Undaria pinnatifida* [18], *Gracilaria gracilis* [19], and *Palmaria palmata* [20]. Furthermore, the well-balanced ratio between *n*-6 and *n*-3 PUFA become these sea vegetables attractive [21], namely in the prevention and mitigation of chronic inflammatory diseases [22]. 

In the literature, it has been reported that some seaweeds contain essential fatty acids—alpha-linolenic acid (ALA; C18:3 *n*-3) and linoleic acid (LA; C18:2 *n*-6) [3,10,23]—that are not synthesized by the human body and need to be obtained through diet [24]. In the human body, essential fatty acids are converted in long-chain eicosapentaenoic (EPA; C20:5 *n*-3) and arachidonic (AA, C20:4 *n*-6) fatty acids that play key roles in inflammatory processes, brain function, cardiovascular disease, obesity and cancer [25,26]. Red seaweeds (*Palmaria palmata* and *Porphyra* sp.) are particularly rich in EPA [3], while AA is mostly found in red and brown seaweeds, such as *Gracilaria gracilis* and *Fucus vesiculosus*, respectively [19,20].

During the last decade, the interest and demand for seaweeds in the Western countries have been growing. Nowadays, there is also a greater offer of seaweed-based ingredients and food products such as, sausages, burgers, bread, pasta, pastries, yogurts, cheeses, etc. The incorporation of seaweeds in foodstuff allows improvement of their nutritional quality, functional properties and shelf life [1]. Seaweeds and seaweed-based foods consumption is suitable for everyone and is in line with the European Green Deal goals and Farm to Fork strategy for a healthy and fully sustainable food system [27]. In this way, the development of the seaweed commercial sector aims to fulfill the needs of new markets and the demands from consumers with special needs (e.g., vegans, vegetarians, elderlies). These trends are boosting new algae-based products for applications in several markets, including human food and nutraceuticals [28].

Each seaweed species has specific nutritional and functional characteristics [10,13,29,30,31]. Seaweed blends, resulting from the mixture of two or more species, allow to obtain in a single product better nutritional, health and functional characteristics, not possible to obtain with a single seaweed. The product segment of seaweed blends is still little explored but is becoming more attractive for new food products and promising for new applications.

Hence, this work aimed to evaluate and compare the biochemical composition, fatty acid profile determined by gas chromatography–mass spectrometry (GC–MS) and the healthy lipids quality indices of five commercially available seaweed blends. The results reported will provide useful information on nutritional and healthy values of these mixtures, highlighting their potential as a sustainable dietary source of PUFA and healthy fats. To the best of our knowledge, this is the first study about the composition of seaweed blends.

## 2. Results and Discussion

In this study, the chemical composition and the nutritional value, including the fatty acid profiles and lipid quality indices of five commercial seaweed blends, produced by the company ALGAplus Ltd. (BLD0N1, BLD0N2, BLD001, BLD005 and BLD006—Table 1), were evaluated.

### 2.1. Chemical Composition

**Moisture:** Seaweed blends moisture content ranged between 10.64 ± 0.23% for sample BLD0N1 and 13.72 ± 0.21% for sample BLD006 (Table 2). Ideally, the moisture level of dehydrated seaweed should be equal to or less than 15% [32]. The results are in agreement with the recommended values. Seaweeds are usually commercialized and consumed as dried biomass and thus with low moisture content, because fresh seaweed is a highly perishable food product. Fresh algal biomass has a moisture content over 80%, deteriorating within a few days after harvest [2]. Drying is a very common process in the industry to preserve biomass for long time periods (3–4 years), since, by decreasing water activity, it retards microbial growth, helps to preserve desirable qualities and still reduces the storage volume [33].

**Ash:** The ash content gave a rough estimate of the total amount of inorganic nutrients present in the food sample. Higher values of ash corresponds to lower organic matter content per biomass amount [34]. All blended samples showed a high content in ash, between a minimum of 22.99 ± 0.29% DW in BLD0N2 and a maximum of 32.10 ± 0.20% DW in BLD0N1 (Table 2), and were within the ranges previously reported for *Alaria esculenta* (21.0% [35]; 24.6% [11]; 33.0% [35]), *Fucus vesiculosus* (20.9% [11]; 21.0% [36]; 25.5% [37]), *Gracilaria gracilis* (24.8% [9]; 28.9% [37]), *Palmaria palmata* (34.0 [12]; 42.2% [11]), *Porphyra* spp. (19.1% [12]; 28.2% [10]), *Ulva rigida* (20.6% [2]; 26.5% [15]; 31.7% [37]) and *Undaria pinnatifida* (31.2% [12]; 34.0% [38]).

**Protein:** Crude protein content was estimated from the analysis of total nitrogen, using the nitrogen–protein conversion factor (F_N-P_) of 6.25, chosen because it is widely applied in the food industry. The highest protein content was obtained for blend BLD005, estimated at 26.61 ± 1.98% DW, while BLD0N1, BLD0N2, BLD001 and BLD006, showed, respectively, protein amounts of 22.27 ± 0.32, 25.97 ± 2.84, 17.79 ± 3.67 and 21.74 ± 3.40% DW (Table 2). In this study, we also applied an F_N-P_ of 5, which is more specific for seaweed, according to Angell et al. [39], since some studies have reported the presence of considerable amounts of non-protein nitrogen in the algal matrix [8,40]. The increasing order of the blends by protein content was in accordance with that observed for an F_N-P_ of 6.25, as follows: BLD001 < BLD006 < BLD0N1 < BLD0N2 < BLD005. The corresponding protein contents were 14.23 ± 2.93, 17.39 ± 2.72, 17.82 ± 0.25, 20.78 ± 2.27 and 21.29 ± 1.58% DW, respectively.

Although the protein contents were different when comparing the five blends, all can be considered a good source of protein (EC no. 1924/2006). The amounts of protein in different seaweeds phyla are established in literature and described as follows: red ≥ green > brown [29]. Accordingly, blend BLD001 showed the lowest protein content, which resembles the amount of protein found in brown seaweeds. In contrast, the protein levels of samples BLD005 and BLD0N2, with an average values of protein content of approximately 26% DW (F_N-P_ = 6.25), were the ones with highest protein content and similar to those reported in red seaweeds. 

The world’s population growth and environmental pressure have led to the need to promote the incorporation of more sustainable protein sources into the Western diet [41]. It is also consensual that individuals that practice moderate to intense physical activity, elderly, pregnant women and lactating women require more protein than the overall population [42,43], generating demand for protein-rich food sources. The seaweed blends analyzed in this study are a sustainable source of protein, alternative to animal or other vegetable sources (e.g., soybean, peas), and have greater nutritional value, suitable for all types of diet and age groups.

**Lipids:** The total lipid content of the five blends was estimated by gravimetry of the lipid extracts. The blend BLD005 (0.55 ± 0.13% DW) recorded the lowest fraction, while the blend BLD0N1 (1.50 ± 0.09% DW) achieved the highest lipid fraction (Table 2). Similar lipid levels were reported in the literature for the seaweed species from wild origins and from species produced in aquaculture in Europe [9,12,15,37,44]. As expected, the total lipid content was low in comparison with microalgae [45], and all blends were considered as low-fat foods (≤3 g/100 g) (EC no. 1924/2006). The lipid level of blend BLD005 was statistically different from the others. The blend BLD005 had the lowest lipid content, with similar values to those described for red seaweed *Palmaria palmata* and *Porphyra* spp. [3,20,38], suggesting that this blend could have great amounts of red seaweeds.

In spite of the low fat content detected, other studies showed that algal lipids are a good source of healthy fatty acids, namely essential fatty acids and *n*-3 PUFA [3,44], that can contribute for diseases prevention and for sustainable and healthy diets. 

**Total carbohydrates:** Total carbohydrates amounts were calculated from the difference of the other parameters (moisture, ash, protein (F_N-P_ = 6.25) and lipids) and ranged between 43.50 ± 1.95% DW in blend BLD005 to 54.73 ± 3.65% DW in blend BLD001 (Table 2), being consistent with the literature for the seaweed species present in the blends [9,37]. The calculated values reflected the sum of nutritionally available carbohydrates (dextrins, starches and sugars) with the dietary fiber [46]. Several studies indicate that most algal polysaccharides are neither digested nor absorbed in the human gastrointestinal system, representing dietary fiber [14,18,37], so the nutritional quality of the analyzed blends could be considered high. Dietary fiber is not an essential component of the human diet, but its deficient intake is a risk factor for gastrointestinal disorders, cardiovascular diseases, obesity and type II diabetes mellitus [26,47]. Considering that many Western citizens are not meeting the recommended daily allowances for dietary fiber [47], the consumption of seaweed blends as food or food ingredients can help achieve the recommended quantities.

### 2.2. Fatty Acid Profiles and Lipid Quality Indicators

#### 2.2.1. Fatty Acid Profiles

Despite the small lipid contents recorded in the five blends, seaweeds are considered an excellent source of UFA, namely PUFA [3,18,44,48]. Fatty acid profiles of the lipid extracts from blends BLD0N1, BLD0N2, BLD001, BLD005 and BLD006 were determined by GC–MS and the content of the identified fatty acids was calculated and expressed in percentage of the total fatty acid methyl esters (FAMEs) (Table 3). The results provide a nutritional evaluation of the composition in fatty acids and their health-related lipid indices, revealed in the five seaweed blends.

A maximum of 33 fatty acids were identified in blend BLD006 and a minimum of 27 fatty acids in blend BLD001. Twenty fatty acids were common to all seaweed blends, including myristic (C14:0), palmitic (C16:0), stearic (C18:0), myristoleic (C14:1), hypogeic (C16:1 *n*-9), palmitoleic (C16:1 *n*-7), oleic (C18:1 *n*-9), cis-vaccenic (C18:1 *n*-7), eicosenoic (C20:1 *n*-9), hexadecatetraenoic (C16:4 *n*-3), linoleic (LA; C18:2 *n*-6), gamma-linolenic (C18:3 *n*-6 GLA), alpha-linolenic (ALA; C18:3 *n*-3), stearidonic (SDA; C18:4 *n*-3), arachidonic (AA; C20:4 *n*-6), eicosatetraenoic (C20:4 *n*-3) and eicosapentaenoic (EPA; C20:5 *n*-3) acids.

Each blend showed a different and specific major fatty acid. The FA C16:0 was the major one in blend BLD0N1 (26.83 ± 0.84%); FA C18:1 *n*-9 was the most abundant in blends BLD006 (22.70 ± 2.23%) and BLD0N2 (34.09 ± 2.90%); and EPA was the dominant fatty acid in blend BLD005 (39.13 ± 5.84%). Exceptionally, blend BLD001 showed two major fatty acids, FA C16:0 (27.33 ± 1.52%) and EPA (26.79 ± 1.37%), with similar relative abundances (*p* > 0.05).

**Figure 1:**
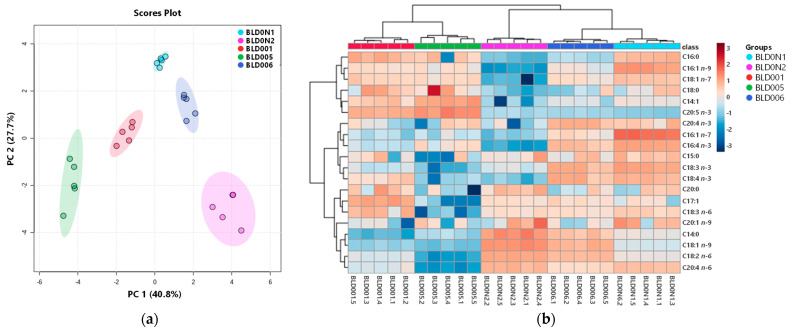
Seaweed blends discrimination, based on the profile of the 20 fatty acids commonly identified in the blends. (**a**) Principal component analysis (PCA) scores plot. (**b**) Heatmap with the clustering dendrogram of blends.

Principal component analysis (PCA) was carried out to visualize similarities between blends and correlations between fatty acids. To reduce the number of descriptors associated with the dataset, while explaining the maximum amount of variability present, only the data of the 20 fatty acids common to the samples analyzed were subjected to the analysis. PCA scores plot (Figure 1a) showed a complete discriminative separation of the macroalgae blends. PCA analysis described 68.5% of the total variance, including principal component 1 (40.8%) and principal component 2 (27.7%). The fatty acids LA (0.34), C18:1 *n*-9 (0.33), AA (0.31), C14:1 (−0.31), C14:0 (0.29) and EPA (−0.29) were the major contributors to the variability observed along the first dimension of the PCA, and the variables with higher contributions to the second dimension included SDA (0.39), C16:1 *n*-7 (0.37), C16:4 *n*-3 (0.35), C16:1 *n*-9 (0.34), ALA (0.33) and C18:1 *n*-7 (0.30). The PCA scores plot evidenced the variability within blends, mainly in BLD005 and BLD0N2. Hierarchical clustering heatmap (Figure 1b) analysis revealed that the five seaweed blends were grouped into two main clusters. Blends BLD001 and BLD005 were clustered together mainly due to their high content of EPA, and the other cluster included blends BLD0N1, BLD0N2 and BLD006, characterized by the presence in greater concentration of AA, LA, C18:1 *n*-9 and C14:0. At the second level of the tree, blends BLD0N1 and BLD006 were more similar to each other, as relative abundances of *n*-3 PUFA species (ALA, SDA, C20:4 *n*-3, C16:4 *n*-3) and C16:1 *n*-7 were higher than in blend BLD0N2.

**Figure 2:**
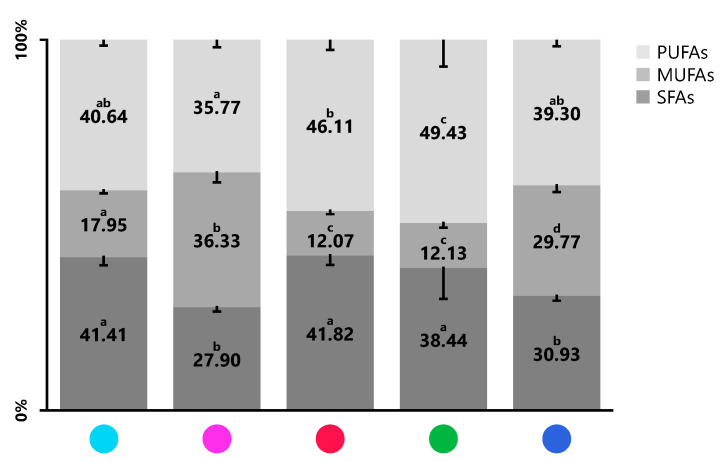
Proportion (%) of saturated fatty acids (SFA), monounsaturated (MUFA) and polyunsaturated (PUFA) identified in the seaweed blends BLD0N1 ●, BLD0N2 ●, BLD001 ●, BLD005 ●, and BLD006 ●. Values are expressed as mean ± SD (n = 5). Different lower-case letters mean significant statistical differences at a 5% level.

For each product, the sum of SFA, MUFA and PUFA were calculated (Figure 2 and Table 3). All the analyzed blends presented amounts of SFA less than 42%, ranging between 27.90 ± 1.27% in blend BLD005 and 41.82 ± 2.54% in blend BLD001. The lipid profiles of BLD006 and BLD0N2 were the richest in terms of UFA; however, blend BLD0N2 had the lowest PUFA content (35.77 ± 2.02%), whilst blends BLD005 (49.43 ± 7.24%) and BLD001 (46.11 ± 2.72%) presented significantly higher amounts of PUFA.

**Saturated fatty acids:** In the five blends, the majority SFA was FA C16:0 (14.34 ± 0.78%–27.33 ± 1.52%), followed by FA C18:0 (6.54 ± 0.77%–12.66 ± 4.73%) and FA C14:0 (2.27 ± 0.16%–6.40 ± 0.45%). Lauric acid (C12:0) was only detected in a trace form (<0.01%) in blends BLD006 and BLD0N2. The higher abundance of FA C16:0 in the seaweed blends was in agreement with that observed in other seaweed biomass [3,18,29,37] while the second most abundant was the FA 18:0. Few studies reported higher relative content of FA C14:0 compared with FA C18:0 in seaweed [3,11,12,18,44], but this was not seen in the present study. High consumption of SFA is positively correlated with increased cholesterol levels, and consequently is associated with increased risk and mortality from cardiovascular disease [49]. However, each SFA is characterized by its metabolic behavior. Long-chain SFA (C14:0, C16:0 and C18:0) are considered thrombogenic factors that promote platelet aggregation [50]. Additionally, FA C14:0 and FA C16:0, together with FA C12:0, favor the adhesion of lipids to circulatory and immune systems cells (pro-atherogenic), while FA C18:0 has anti-atherogenic properties by lowering LDL cholesterol and raising HDL cholesterol, surpassing some MUFA in terms of health benefits [51]. From a nutritional and health point of view, the mixture of two or more species allowed the final products to have a higher content of C18:0 (thrombogenic but anti-atherogenic) than C14:0 (thrombogenic and pro-atherogenic), a notorious fact in blends BLD001, BLD005 and BLD0N1, the three with the highest relative percentage of SFA.

**Monounsaturated fatty acids:** MUFA fractions had the major contribution of FA C18:1 *n*-7 and FA C18:1 *n*-9, the latter being significantly and abundantly detected in blends BLD0N2 (34.09 ± 2.90%) and BLD006 (22.70 ± 2.23%). Another MUFA, present in all mixtures above 1%, was the FA C16:1 *n*-7. Similar results were found in the literature for the seaweed species that constitute the blends [3,37]. In blends BLD0N2 and BLD006, the most abundant MUFA was the FA C18:1 *n*-9, that was also the most abundant MUFA in brown seaweed *Fucus vesiculosus* [3,12], an ingredient of these blends. Despite the scarce evidence regarding the impact of MUFA on health, it is suggested that MUFA, integrated into the plant food matrix—which is the case of the samples under study—attenuate the risk of mortality from all causes, as opposed to animal sources [50]. In particular, FA C18:1, the major fatty acid in olive oil (>70% of the total fatty acids), which was appreciably detected in blends BLD0N2 and BLD006, is associated with the well-known health-promoting properties present in olive oil [52].

**Polyunsaturated fatty acids:** PUFA present in the studied blends occur mainly in the form of *n*-6 FA (C16:2, C18:2, C18:3, C20:2, C20:3 and C20:4) and *n*-3 PUFA (C16:3, C18:3, C18:4, C20:3, C20:4, C20:5 and C22:5). Of the 19 PUFA detected, only 8 were common to all blends, among which the essential fatty acids LA and ALA, the SDA and the long-chain PUFA (LC-PUFA) AA and EPA stand out. Due to their nutritional importance, these five PUFA will be discussed below.

The two essential fatty acids LA and ALA were detected in all blends at levels not higher than 7%. The relative abundance of LA was significantly different between all blends (*p* < 0.05) and, regarding ALA values, similarities were observed between blends BLD0N1 (6.14 ± 0.22%) vs. BLD006 (5.59 ± 0.56%) and BLD0N2 (3.12 ± 0.23%) vs. BLD001 (2.91 ± 0.26%) (*p* > 0.05).

The SDA (C18:4 *n*-3), which is a precursor of EPA, ranged between 3.79 ± 0.44% in blend BLD0N2 and 8.69 ± 0.31% in blend BLD0N1. Blend BLD0N1 was similar to BLD006 (8.64 ± 0.94%) (*p* > 0.05), which also did not present significant differences between BLD001 (7.04 ± 0.71%) (*p* > 0.05).

The maximum values for AA were observed in samples BLD0N1 (10.75 ± 0.36%) and BLD0N2 (12.31 ± 0.84%). Among the PUFA identified in each blend, this was the most abundant in both BLD0N1 and BLD0N2. All the five mixtures showed significant differences from each other (*p* > 0.05). Blend BLD005 recorded the lowest relative amount of AA (1.03 ± 0.16%).

Finally, EPA was the major contributor to the sum of all PUFA identified in blends BLD005 (39.13 ± 5.84%) and BLD001 (26.79 ± 1.37%), and its contribution was also appreciable, but with much less expression, in blends BLD0N2 (5.13 ± 0.50%), BLD006 (4.77 ± 0.26%) and BLD0N1 (3.70 ± 0.22%). Blends BLD005 and BLD001 were significantly similar to each other (*p* < 0.05), but different from the others (*p* < 0.05).

Figure 2 illustrates that PUFA account for approximately 40% of the relative abundance of the identified fatty acids. LA and ALA are essential fatty acids that the human body is unable to synthesize, so they must be obtained through the diet [22]. Both are present in the five blends in significantly different proportions (*p* < 0.05). In green and red seaweeds, LA represents no more than 3% of the total FAMEs, while in brown seaweeds, the average value is 8% [3,11,48]. Literature reports lower relative abundances of ALA in Rhodophyta (1–3%), followed by Ochrophyta (5–7%, 12% in *Undaria pinnatifida*) and, lastly, in Chlorophyta (11–15%) [3,11,48]. Humans can synthesize *n*-6 LC-PUFA, like AA, from LA and *n*-3 LC-PUFA, as EPA and docosahexaenoic acid (DHA; C22:6 *n*-3), from ALA [24]. AA, EPA and DHA originate lipid mediators that are important in immune regulation and the inflammatory process, as they are precursors of pro- and anti-inflammatory mediators [22,24]. Only AA and EPA were identified in all blends, as verified in the isolated algae species that constitute the blends [3,12,18]. AA is a specific PUFA found in greater abundance in *Fucus vesiculosus* and *Gracilaria gracilis* [3,12], which was reflected in the content of AA in blends BLD0N1 and BLD0N2 that contain those seaweed species. AA is needed in the body for an adequate inflammatory response, it also helps regulate neuronal activity and benefits ocular health [22,24]. Nevertheless, their consumption should contribute to a total intake of *n*-6 PUFA equivalent to or less than that of *n*-3 PUFA [22]. In turn, EPA is the characteristic fatty acid of some Rhodophyta, such as *Palmaria palmata* and *Porphyra* spp. [3,44]. With about 40% of the total FAMEs, this was the main fatty acid of sample BLD005, which seems to be very rich in red seaweeds. In BLD001, EPA represented approximately 27% of the total FAMEs and 58% of the PUFA fraction. These two products (BLD001 and BLD005) can therefore be potential sources of *n*-3 LC-PUFA, supporting the current health recommendation that aims to increase the daily consumption of *n*-3 LC-PUFA [49]. EPA is one of the most important fatty acids for human health. In addition to being anti-inflammatory, it acts as a regulator of the circulatory system, in slimming or muscle definition diets, it prevents protein catabolism and acts beneficially at the neuronal level [24]. Some evidence suggests that SDA is more easily converted by humans to EPA than ALA; however, its dietary sources are limited [53]. The presence of SDA in the five mixtures is in agreement with other investigations in several seaweed species [3,44]. Particularly, blends BLD006 and BLD0N1, where the relative abundance of EPA was low, can help to increase the daily intake of SDA.

Independently of the selected mixture, these seaweed blends are a natural source of PUFA, particularly *n*-3 PUFA, being an excellent alternative to fish (including crustaceans and shellfish), compatible with vegetarian and vegan diets [54].

#### 2.2.2. Lipid Quality Indicators

The lipid quality of the total lipid extracts of seaweed blends BLD0N1, BLD0N2, BLD001, BLD005 and BLD006 was evaluated, based on the nutritional and health indicators UFA:SFA, PUFA:SFA, *n*-6:*n*-3 PUFA, atherogenicity index (AI) and thrombogenicity index (TI) (Table 4). The indices AI and TI are theoretical calculations and are the most commonly used indices to assess the nutritional value of fatty acids and to evaluate the potential of food to contribute to the prevention of cardiovascular diseases [55].

For the five blends under investigation, UFA:SFA ratios between 2.59 ± 0.16 (BLD0N2) and 1.40 ± 0.15 (BLD001) were found, and PUFA:SFA ratios ranged from 0.99 ± 0.09 (BLD0N1) to 1.37 ± 0.48 (BLD005). Except for blend BLD0N2, *n*-6:*n*-3 PUFA ratios below 1 were recorded in the remaining blends. Regarding AI and TI, the lowest values were calculated in blends BLD005 for AI (0.23 ± 0.09) and BLD006 for TI (0.57 ± 0.17), while the highest indices were determined in blend BLD0N1 (AI: 0.72 ± 0.04; TI: 0.45 ± 0.04). Significant differences between the seaweed blends were revealed for UFA:SFA ratio, *n*-6:*n*-3 PUFA ratio, AI and TI. Table 5 shows the calculated values reported in the literature for the seaweed species present in each blend, to compare with the values obtained for the five seaweed blends. 

The PUFA:SFA ratio allows assessment of the influence of diet on cardiovascular health [55]. The UFA:SFA ratio is a complementary parameter since the consumption of MUFA is also related to an improvement in serum cholesterol levels and the maintenance of cell function, helping to prevent cardiovascular diseases [56,57]. Both ratios were calculated for all blends and indicate that seaweeds blends have beneficial proportions of UFA and PUFA for human health (≥1). Some of the calculated values for the seaweed blends showed better results than those reported in the literature for each species (Table 5).

The balance between *n*-6 (pro-inflammatory) and *n*-3 (anti-inflammatory) fatty acids is a nutritional factor of great relevance [58]. The values found for the studied blends are within the range described in the literature for all species present in the mixtures and are considered appropriate for healthy diets (Table 5). The *n*-6:*n*-3 PUFA ratio affects the regulation of metabolic functions and the development of associated syndromes, such as insulin resistance, adiposity and inflammation [22]. Although the ideal dietary intake ratio of *n*-6:*n*-3 PUFA was set at 1-4:1, the Western diet is characterized by an unbalanced consumption of *n*-6 PUFA, with as estimated ration of 15:1, which may contribute to the development and pathogenesis of the cardiovascular diseases, cancer, inflammation and autoimmune diseases [24]. Hence, recommendation for healthy diets prioritize the ingestion of foods with *n*-6:*n*-3 PUFA ≤ 1, as shown for blends BLD0N1, BLD001, BLD005 and BLD006.

Additionally, the AI and TI values should preferably be less than 1, and the lower the values the healthier the seaweed blend can be considered [59]. These are indicators of the probability that a given food, based on its fatty acid composition, will contribute to the incidence of physio-pathological events, such as atheroma on the inner walls of arteries and the occurrence of thrombi [55]. The results reported were similar to those described for isolated species (Table 5) and marine products, such as other seaweed, fish and shellfish [55], thus, they are good sources of healthy lipids. Based on the above, blend BLD0N1 was negatively evidenced, with the worst values in both indexes.

Overall, the evaluation of fatty acid profile provides evidence of the proper choice of seaweed species for the blend formulation, given its high PUFA content, low *n*-6:*n*-3 PUFA ratio and small AI and TI values. This strategy could be useful to improve the human diet quality, preventing inflammatory, cardiovascular and nervous system disorders. It is important to clarify that the impact of a given food on human health is not governed by its fatty acid composition, but by the set of all its nutritional parameters.

## 3. Materials and Methods

### 3.1. Seaweed Blends Samples

Five seaweed blends (BLD0N1, BLD0N2, BLD001, BLD005 and BLD006) were provided by the company ALGAplus Ltd. (Ílhavo, Portugal), a seaweed farmer in a land-based integrated multi-trophic aquaculture system (IMTA) in Portugal (production site in Ria de Aveiro, Portugal, 40°36′43″ N, 8°40′43″ W). Each blend consisted of two to four dried and ground seaweed species produced under EU organic standards (Table 1). According to the supplier specifications, seaweeds were oven-dried at 30 °C, up to a moisture content less than 15%, and ground up to 25 mm.

### 3.2. Chemical Composition Determination

#### 3.2.1. Moisture and Ash

For moisture content determination, the samples (250 mg × 5 replicates (n = 5)) were dried in crucibles on an oven at 105 °C for 8 h. Moisture content (%) was obtained by Equation (1), as follows:

(1) $$\mathrm{Moisture}\;\mathrm{content}\;\;\left(\%\right)=\frac{\mathrm{Initial}\;\mathrm{sample}\;\mathrm{weight}\;(g)\;-\;\mathrm{Dry}\;\mathrm{sample}\;\mathrm{weight}\;(g)}{\mathrm{Initial}\;\mathrm{sample}\;\mathrm{weight}\;(g)}\times 100$$

Ash content was quantified gravimetrically after direct incineration at 575 °C for 22 h of the dry sample (250 mg × 4 replicates (n = 4)) on a muffle furnace equipped with a ramp and landing program (Nabertherm GmbH, Lilienthal, Osterholz, Germany), in accordance with that described by Van Wychen and Laurens [60]. Ash content (% DW) was calculated using Equation (2), as follows:

(2) $$\mathrm{Ash}\;\mathrm{content}\;\left(\%\;\mathrm{DW}\right)=\frac{\mathrm{Ash}\;\mathrm{weigh}\;(g)\;}{\mathrm{Dry}\;\mathrm{sample}\;\mathrm{weight}\;(g)}\times 100$$

#### 3.2.2. Crude Protein

Crude protein content was indirectly estimated multiplying the elemental nitrogen content of the sample (2 mg × 3 replicates (n = 3)), previously determined by thermal conductivity using a Leco TruSpec 630-200-200 CNHS analyzer (St. Joseph, MI, USA), by 2 nitrogen–protein conversion factors of 6.25 [34] and 5 [39]. The results were expressed in % of dry weight of sample (DW).

#### 3.2.3. Lipid Extraction

Lipid content was determined gravimetrically after total lipids extraction was per-formed according to a modified Bligh and Dyer method, as previously described in studies carried out by the research team [3,61]. The sample (250 mg × 5 replicates (n = 5)) was mixed with 3.75 mL of a solvent mixture of methanol and dichloromethane (2:1, *v*/*v*) in a glass PYREX tube, followed by homogenization in a vortex for 2 min and ultrasound bath for 1 min. After incubation on ice on a rocking platform shaker (Stuart equipment, Bibby Scientific, UK) for 2 h, the suspension was centrifuged (Selecta JP Mixtasel, Abrera, Barcelona, Spain) for 10 min at 2000 rpm, and the organic phase was collected. To promote phase separation and wash the lipid extract, 1.25 mL of dichloromethane and 2.25 mL of Milli-Q water were added to the collected organic phase, centrifuged for 10 min at 2000 rpm, and the lower organic phase was recovered. Re-extraction steps using the same solvent proportions were repeated two more times. The organic phases collected were dried under a stream of nitrogen. Extracts were weighed to determine lipid content and stored at −20 °C. The results were expressed in % of dry weight of sample (DW).

#### 3.2.4. Fatty Acids Profiling by Gas Chromatography–Mass Spectrometry

Fatty acid profiling was performed by gas chromatography–mass spectrometry (GC–MS), as described in previous studies [3,61]. Fatty acid methyl esters (FAMEs) were prepared from blends of total lipid extracts by adding a methanolic solution of potassium hydroxide (2.0 M). A hexane solution containing FAMEs (2 μL × 5 replicates (n = 5)) was injected in a GC–MS (Agilent Technologies 6890 N Network, Santa Clara, CA, USA) equipped with a DB-FFAP column (30 m long, 0.32 mm internal diameter, and 0.25 μm film thickness; J and W Scientific, Folsom, CA, USA). The GC equipment was connected to an Agilent 5977B Mass Selective Detector operating with an electron impact mode at 70 eV and scanning range of 50–550 *m*/*z* in a one second cycle in full scan mode acquisition. The oven temperature was programmed from an initial temperature of 80 °C for 2 min, a linear increase to 160 °C at 25 °C min^−1^, followed by linear increase to 210 °C at 2 °C min^−1^, then to 250 °C at 20 °C min^−1^, standing at 250 °C for 20 min. The injector and detector temperatures were 220 °C and 230 °C, respectively. Helium was the carrier gas at a flow rate of 1.4 mL min^−1^. The software GCMS5977B/Enhanced MassHunter was used for data acquisition. The acquired data were analyzed using the qualitative data analysis software Agilent MassHunter Qualitative Analysis 10.0, and FA identification was performed by MS spectrum comparison with the chemical database NIST library and confirmed with the literature. The relative amounts of each FA were calculated by the percent relative area method with proper normalization using internal standard methyl nonadecanoate (C19:0, Sigma-Aldrich, St. Louis, MO, USA), considering the sum of all relative areas of identified fatty acids.

Nutritional quality of the lipid fraction was evaluated based on the fatty acid profile, through the UFA:SFA, PUFA:SFA and PUFA *n*-6:*n*-3 ratios, and by the atherogenicity (Equation (3) AI) and thrombogenicity (Equation (4) TI) indices. The indices of AI and TI were calculated according to the equations reviewed by Chen and Liu [55], listed below.

(3) $$\mathrm{AI}=\frac{C12:0+(4\;\times \;C14:0)+C16:0\;}{\sum \mathrm{UFA}}$$

(4) $$\mathrm{TI}=\frac{C14:0+C16:0+C18:0\;}{\left(0.5\;\times \;\sum \mathrm{MUFA}\right)+\left(0.5\;\times \;\sum n-6\right)+\left(3\;\times \;\sum n-3\right)+(\mathrm{PUFA}\;n-6:n-3)}$$

#### 3.2.5. Total Carbohydrates

The total carbohydrate content of each sample was calculated by difference with the remaining parameters of the nutritional profile [34]. The results were expressed in % of dry weight of sample (DW).

### 3.3. Statistical Analysis

All the experimental data are shown as mean ± standard deviation. Univariate statistical analysis was carried out using GraphPad Prism 9 software (GraphPad Software Inc., San Diego, CA, USA). Tests of normality (Shapiro–Wilk test or Kolmogorov–Smirnov test) and homogeneity of variance (Brown–Forsythe test or Bartlett test) were applied before the analysis. Kruskal–Wallis test, followed by Dunn’s post hoc comparisons, were performed in the absence of normality. One-way ANOVA followed by Tukey’s post hoc comparison was applied when checking for normality. Differences between means at the 5% (*p* < 0.05) level were considered significant.

After normalization (log transformation and autoscaling) of the data, principal components analysis (PCA) and heatmap were performed using MetaboAnalyst 5.0 (Xia Lab @ McGill, Quebec, Canada; http://www.metaboanalyst.ca accessed 1 October 2021) to analyze dissimilarities among the samples in terms of fatty acids components [62]. 

## 4. Conclusions

This study evaluated and compared the nutritional values of five commercially available seaweed blends, sustainably produced in Europe, with emphasis on the lipid fraction. To our knowledge, this is the first report assessing the composition of seaweed blends.

The blends were characterized by high ash levels, corresponding to high mineral loads, and shown to be good sources of protein of vegetable origin with low lipid contents. Lipidomic analysis based on GC–MS methodology showed that each blend has a characteristic fatty acid profile. In the five blends, the most abundant fatty acids were unsaturated, including *n*-3 PUFA. The nutritional and healthy indicators of the blends showed well-balanced *n*-6:*n*-3 ratios, with the AI and TI indices suggesting that they have the potential as key ingredients for sustainable and healthy diets contributing to the prevention and treatment of cardiovascular and inflammatory diseases. The blends can contribute to overcoming *n*-3 PUFA deficiency and replace fish oil in diets of Western populations and at the same time, can aid in the avoidance of the overexploitation of fish and shellfish. 

These seaweed blends can be considered natural sources of valuable nutrients for human health, with a relevant role in the prevention of chronic and inflammatory diseases. Although our results indicate the presence of bioactive lipids, it is important to evaluate bioactive activities to apply these products in functional food. Further investigations must be carried out to prove the health-promoting role of the consumption of the seaweed blends in various diseases, including NCDs. 

## Figures and Tables

**Table 1:** Seaweed blends studied.

Blend Code	List of Ingredients on the Product Label
BLD0N1	*Ulva rigida*, *Porphyra* spp., *Alaria esculenta* and *Gracilaria gracilis*
BLD0N2	*Fucus vesiculosus*, *Gracilaria gracilis* and *Porphyra* spp.
BLD001	*Palmaria palmata*, *Ulva rigida*, *Undaria pinnatifida* and *Porphyra* spp.
BLD005	*Palmaria palmata*, *Ulva rigida* and *Porphyra* spp.
BLD006	*Fucus vesiculosus* and *Ulva rigida*

**Table 2:** Chemical composition (moisture, ash, protein, lipids and total carbohydrates) of the seaweed blends BLD0N1, BLD0N2, BLD001, BLD005 and BLD006. Values are expressed as the mean ± SD (^1^ n = 5; ^2^ n = 4; ^3^ n = 3).

Composition	BLD0N1	BLD0N2	BLD001	BLD005	BLD006
Moisture ^1^ (%)	10.64 ± 0.23 ^a^	11.76 ± 0.22 ^b^	13.45 ± 0.31 ^c^	11.49 ± 0.18 ^b^	13.72 ± 0.21 ^c^
Ash ^2^ (% DW)	32.10 ± 0.20 ^a^	22.99 ± 0.29 ^b^	26.43 ± 0.79 ^c^	29.46 ± 0.36 ^d^	26.47 ± 0.41 ^c^
Protein ^3^ (% DW)	22.27 ± 0.32 ^ab^	25.97 ± 2.84 ^a^	17.79 ± 3.67 ^b^	26.61 ± 1.98 ^a^	21.74 ± 3.40 ^ab^
Lipids ^1^ (% DW)	01.50 ± 0.09 ^a^	01.02 ± 0.12 ^b^	01.03 ± 0.05 ^b^	00.55 ± 0.13 ^c^	01.08 ± 0.08 ^b^
Total carbohydrates ^1^ (% DW)	44.17 ± 0.36 ^a^	50.16 ± 2.89 ^b^	54.73 ± 3.65 ^b^	43.50 ± 1.95 ^a^	50.48 ± 3.46 ^b^

^abcd^—means in the same row with different letters are significantly different (*p* < 0.05). DW—dry weight.

**Table 3:** Fatty acid profile of the studied seaweed blends determined by GC–MS by analysis of fatty acid methyl esters (FAMEs), expressed in relative abundance (%). Values are expressed as the mean ± SD (n = 5).

Fatty Acids	BLD0N1	BLD0N2	BLD001	BLD005	BLD006
C12:0	–	tr	–	–	tr
C14:0	3.79 ± 0.11 ^a^	6.40 ± 0.45 ^b^	2.27 ± 0.16 ^c^	2.95 ± 0.22 ^d^	5.05 ± 0.27 ^e^
C15:0	0.37 ± 0.03 ^a^	0.32 ± 0.06 ^ab^	0.29 ± 0.07 ^ab^	0.20 ± 0.09 ^b^	0.32 ± 0.05 ^ab^
C16:0	26.83 ± 0.84 ^a^	14.34 ± 0.78 ^b^	27.33 ± 1.52 ^a^	22.38 ± 6.40 ^ab^	17.22 ± 0.56 ^ab^
C17:0	–	0.12 ± 0.02	0.17 ± 0.08	0.12 ± 0.07	0.14 ± 0.02
C18:0	9.88 ± 1.78 ^ab^	6.54 ± 0.77 ^b^	11.48 ± 2.04 ^a^	12.66 ± 4.73 ^a^	8.02 ± 1.75 ^ab^
C20:0	0.12 ± 0.06 ^ab^	0.17 ± 0.04 ^b^	0.16 ± 0.07 ^b^	0.06 ± 0.04 ^a^	0.09 ± 0.04 ^ab^
C22:0	0.38 ± 0.26	–	0.11 ± 0.03	0.07 ± 0.05	0.09 ± 0.05
C24:0	0.05 ± 0.04	–	–	–	–
∑SFA	41.41 ± 2.28 ^a^	27.90 ± 1.27 ^b^	41.82 ± 2.54 ^a^	38.44 ± 8.41 ^a^	30.93 ± 1.34 ^b^
C14:1	0.21 ± 0.02 ^abc^	0.05 ± 0.04 ^c^	0.46 ± 0.14 ^ab^	0.96 ± 0.14 ^a^	0.15 ± 0.05 ^bc^
C16:1 *n*-9	1.79 ± 0.17 ^a^	0.16 ± 0.02 ^b^	0.84 ± 0.07 ^c^	0.78 ± 0.14 ^c^	0.64 ± 0.10 ^c^
C16:1 *n*-7	2.85 ± 0.08 ^a^	1.10 ± 0.11 ^b^	1.24 ± 0.14 ^b^	1.32 ± 0.15 ^b^	1.70 ± 0.12 ^c^
C16:1 *n*-5	–	tr	tr	0.38 ± 0.23	0.07 ± 0.03
C17:1	0.13 ± 0.07 ^ab^	0.18 ± 0.02 ^ab^	0.55 ± 0.08 ^a^	tr ^b^	0.19 ± 0.05 ^ab^
C18:1 *n*-9	6.71 ± 0.27 ^a^	34.09 ± 2.90 ^b^	3.62 ± 0.36 ^c^	2.68 ± 0.32 ^d^	22.70 ± 2.23 ^e^
C18:1 *n*-7	6.01 ± 0.28 ^a^	0.47 ± 0.24 ^b^	4.80 ± 0.65 ^c^	4.16 ± 0.68 ^cd^	3.65 ± 0.64 ^d^
C20:1 *n*-9	0.26 ± 0.07 ^a^	0.25 ± 0.11 ^a^	0.14 ± 0.05 ^a^	0.17 ± 0.06 ^a^	0.16 ± 0.04 ^a^
C22:1	–	–	–	0.10 ± 0.05	–
C24:1 *n*-9	–	–	0.38 ± 0.35	1.56 ± 0.22	0.50 ± 0.10
∑MUFA	17.95 ± 0.82 ^a^	36.33 ± 2.72 ^b^	12.07 ± 0.95 ^c^	12.13 ± 1.25 ^c^	29.77 ± 1.82 ^d^
C16:2 *n*-6	0.41 ± 0.01	0.15 ± 0.10	–	–	0.23 ± 0.02
C16:3 *n*-4	0.33 ± 0.04	–	–	–	0.13 ± 0.02
C16:3 *n*-3	–	–	–	–	0.10 ± 0.02
C16:4 *n*-3	4.40 ± 0.16 ^a^	0.19 ± 0.04 ^b^	0.71 ± 0.12 ^b^	1.57 ± 0.52 ^c^	2.84 ± 0.55 ^d^
C16:4 *n*-1	0.40 ± 0.04	–	–	0.07 ± 0.03	0.18 ± 0.02
C18:2	–	–	0.08 ± 0.06	0.47 ± 0.03	–
C18:2 *n*-6 LA	3.33 ± 0.13 ^a^	8.26 ± 0.12 ^b^	2.80 ± 0.16 ^c^	0.83 ± 0.14 ^d^	6.74 ± 0.21 ^e^
C18:3 *n*-6 GLA	0.44 ± 0.03 ^ac^	0.38 ± 0.03 ^ac^	0.51 ± 0.13 ^a^	0.12 ± 0.06 ^b^	0.38 ± 0.02 ^c^
C18:3 *n*-3 ALA	6.14 ± 0.22 ^a^	3.12 ± 0.23 ^b^	2.91 ± 0.26 ^b^	1.72 ± 0.46 ^c^	5.59 ± 0.56 ^a^
C18:4 *n*-3 SDA	8.69 ± 0.31 ^a^	3.79 ± 0.44 ^b^	7.04 ± 0.71 ^ab^	3.89 ± 0.97 ^b^	8.64 ± 0.94 ^a^
C20:2 *n*-6	0.07 ± 0.03	0.57 ± 0.04	–	–	0.37 ± 0.10
C20:3	–	0.63 ± 0.25	–	–	0.44 ± 0.21
C20:3 *n*-6	0.94 ± 0.25	0.90 ± 0.31	0.30 ± 0.08	–	0.51 ± 0.07
C20:3 *n*-3	–	tr	–	–	–
C20:4	–	0.07 ± 0.04	–	–	–
C20:4 *n*-6 AA	10.75 ± 0.36 ^a^	12.31 ± 0.84 ^b^	4.55 ± 0.67 ^c^	1.03 ± 0.16 ^d^	7.54 ± 0.49 ^e^
C20:4 *n*-3	0.40 ± 0.05 ^ac^	0.25 ± 0.08 ^b^	0.30 ± 0.06 ^bc^	0.32 ± 0.12 ^bc^	0.45 ± 0.03 ^a^
C20:5 *n*-3 EPA	3.70 ± 0.22 ^a^	5.13 ± 0.50 ^a^	26.79 ± 1.37 ^b^	39.13 ± 5.84 ^c^	4.77 ± 0.26 ^a^
C22:5 *n*-3	0.64 ± 0.10	–	0.13 ± 0.03	0.28 ± 0.12	0.38 ± 0.10
∑PUFA	40.64 ± 1.54 ^ab^	35.77 ± 2.02 ^a^	46.11 ± 2.72 ^b^	49.43 ± 7.24 ^c^	39.30 ± 1.73 ^ab^

^abcde^—means in the same row with different letters are significantly different (*p* < 0.05). tr—traces.

**Table 4:** Lipid quality indicators of seaweed blends BLD0N1, BLD0N2, BLD001, BLD005 and BLD006. Values are expressed as the mean ± SD (n = 5).

Indicators	BLD0N1	BLD0N2	BLD001	BLD005	BLD006
UFA:SFA	1.42 ± 0.13 ^a^	2.59 ± 0.16 ^b^	1.40 ± 0.15 ^a^	1.70 ± 0.58 ^a^	2.24 ± 0.14 ^b^
PUFA:SFA	0.99 ± 0.09 ^a^	1.28 ± 0.08 ^a^	1.11 ± 0.13 ^a^	1.37 ± 0.48 ^a^	1.27 ± 0.09 ^a^
*n*-6:*n*-3 PUFA	0.67 ± 0.01 ^ab^	1.81 ± 0.11 ^a^	0.22 ± 0.02 ^b^	0.04 ± 0.01 ^b^	0.70 ± 0.10 ^ab^
AI	0.72 ± 0.04 ^a^	0.55 ± 0.03 ^b^	0.63 ± 0.06 ^ab^	0.57 ± 0.17 ^ab^	0.54 ± 0.01 ^b^
TI	0.45 ± 0.04 ^a^	0.40 ± 0.02 ^ac^	0.32 ± 0.03 ^bc^	0.23 ± 0.09 ^b^	0.33 ± 0.03 ^c^

^abc^—means in the same row with different letters are significantly different (*p* < 0.05).

**Table 5:** Lipid quality indicators calculated for the seaweed species that make up the blends under study.

Indicators	*Alaria esculenta*		*Fucus vesiculosus*		*Undaria pinnatifida*		*Gracilaria gracilis*		*Palmaria palmata*		*Porphyra* spp.		*Ulva rigida*	
UFA:SFA	2.42	[29]	1.36 1.77, 2.11 2.85	[37] [48] [3]	3.90 *	[12]	0.78 1.87	[37] [3]	1.44	[3]	1.71	[3]	1.13 3.17	[37] [3]
PUFA:SFA	1.62	[29]	0.94, 1.26 1.96	[48] [3]	3.39 *	[12]	1.51	[3]	1.32	[3]	1.09	[3]	2.63	[3]
*n*-6:*n*-3 PUFA	0.90	[29]	0.40 1.21 1.20, 1.95	[37] [3] [48]	0.49	[12]	0.22 2.48	[3] [37]	0.02	[3]	0.44	[3]	0.03 0.07 0.37	[3] [15] [37]
AI	0.45	[29]	0.66 0.74, 0.90	[3] [48]	0.13 *	[12]	0.61	[3]	0.80	[3]	0.96	[3]	0.27	[3]
TI	0.23	[29]	0.26	[3]	0.37 *	[12]	0.49	[3]	0.20	[3]	0.41	[3]	0.10	[3]

* Indicators calculated from the fatty acid composition of the total lipid extracts found in the literature.
